# Supplementary material for: Assessment of the Molecular Heterogeneity of E-Cadherin Expression in Invasive Lobular Breast Cancer
Source: Cancers (Basel). 2022 Jan 7;14(2):295. doi: 10.3390/cancers14020295 (PMC8773871; doi:10.3390/cancers14020295)
Supplement: Supplementary file 1 [file cancers-14-00295-s001.zip › 1488067_FigureS1_6.pdf]

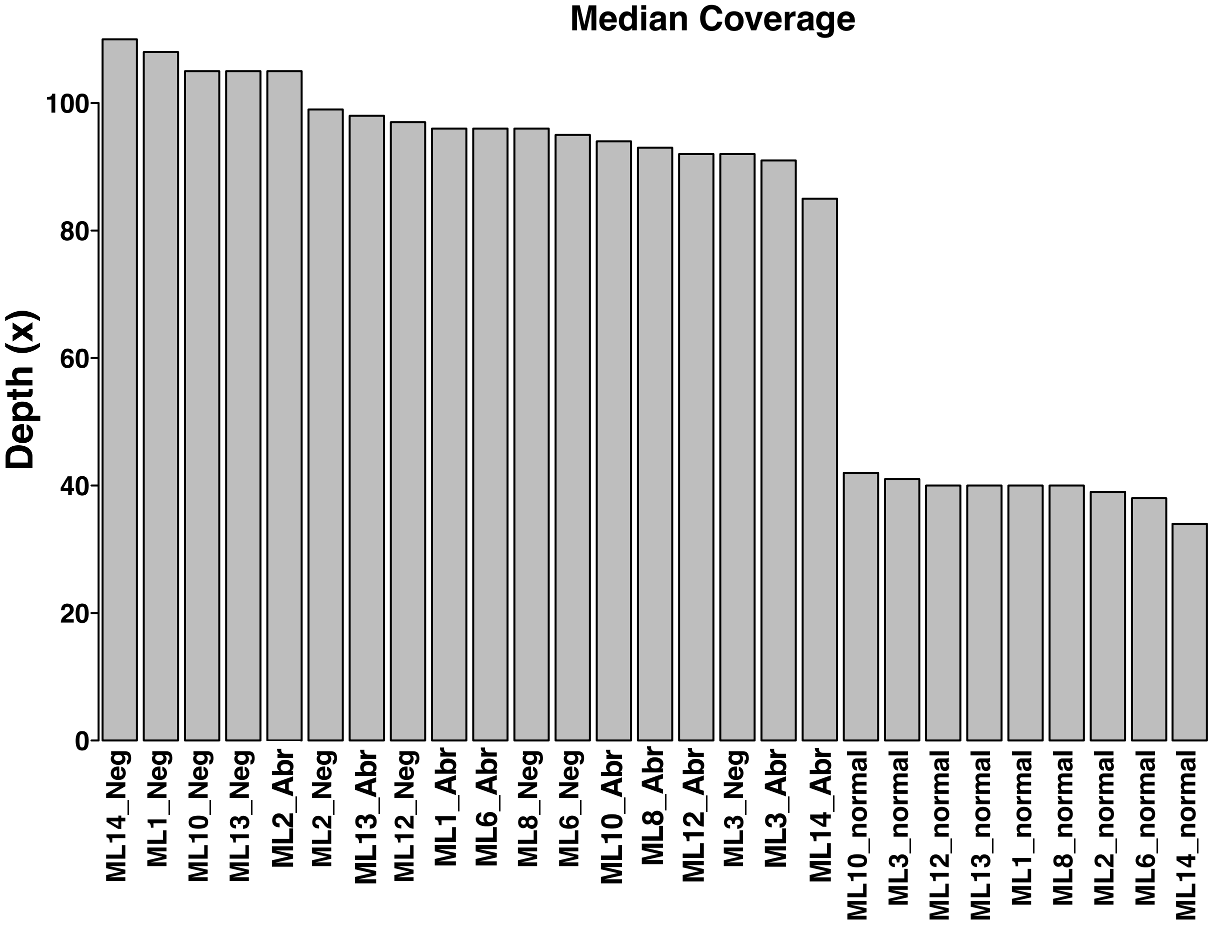

Supplementary Figure 1

Supplementary Figure 2

ML1 Abr

Ploidy: 2.30, aberrant cell fraction: 24%, goodness of fit: 94.4%

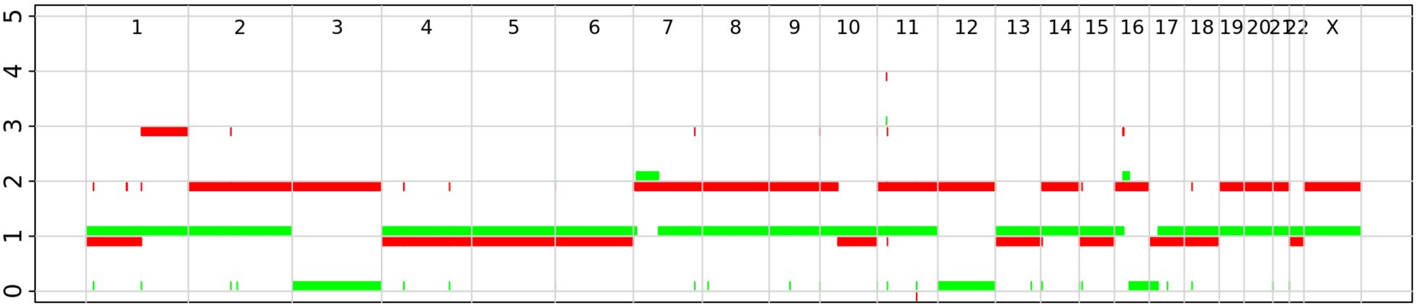

ML1 Neg

Ploidy: 1.99, aberrant cell fraction: 12%, goodness of fit: 68.5%, non-aberrant

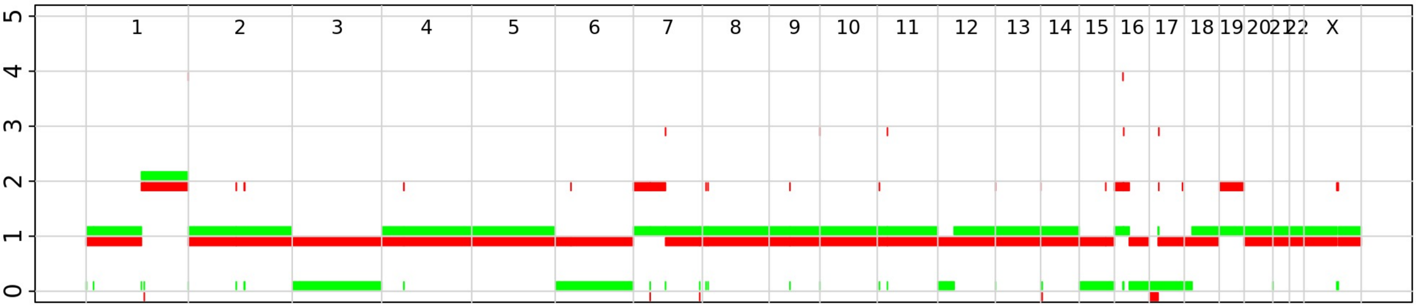

ML2 Abr

Ploidy: 2.01, aberrant cell fraction: 41%, goodness of fit: 98.4%

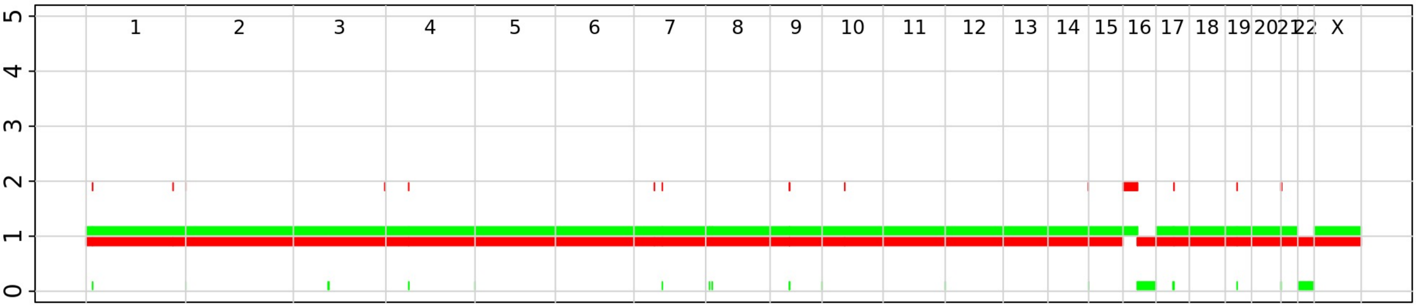

ML2 Neg

Ploidy: 2.01, aberrant cell fraction: 34%, goodness of fit: 95.5%

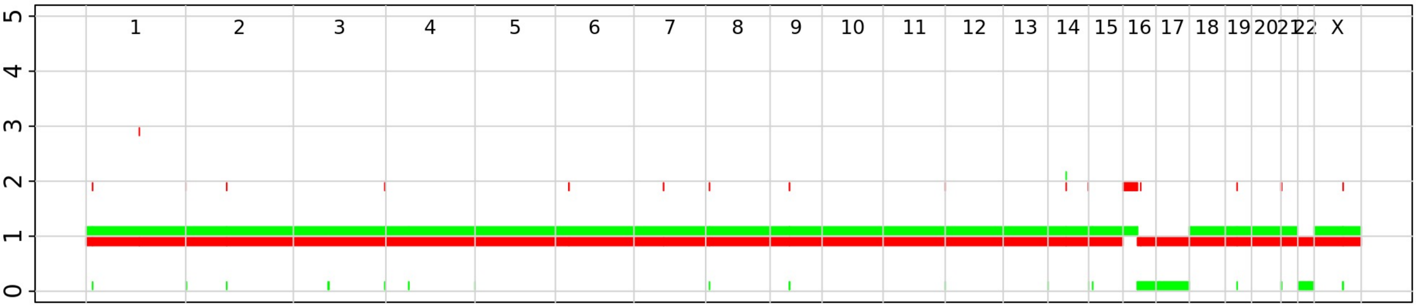

ML3 Abr

Ploidy: 1.99, aberrant cell fraction: 44%, goodness of fit: 98.1%

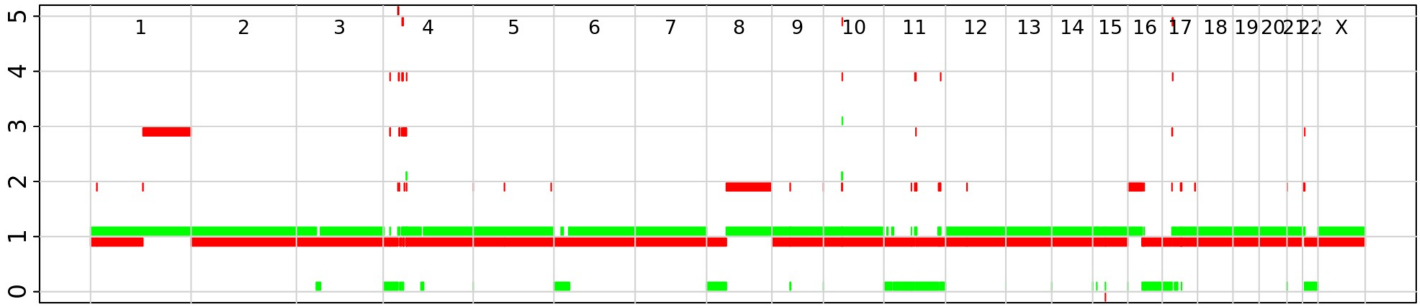

ML3 Neg

Ploidy: 2.00, aberrant cell fraction: 53%, goodness of fit: 98.3%

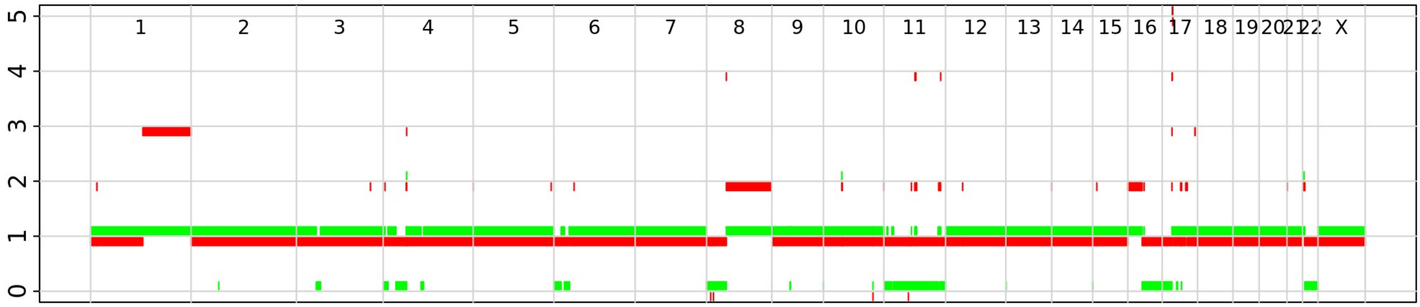

ML6 Abr

Ploidy: 4.26, aberrant cell fraction: 22%, goodness of fit: 98.2%

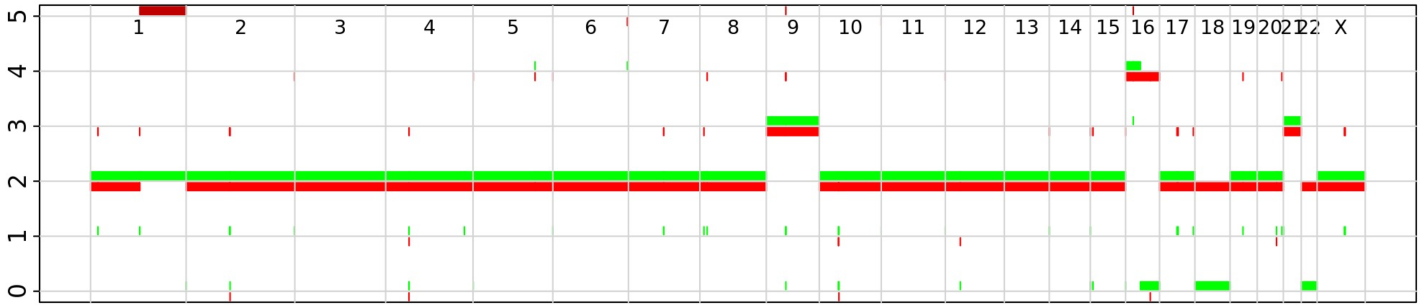

ML6 Neg

Ploidy: 2.13, aberrant cell fraction: 33%, goodness of fit: 94.6%

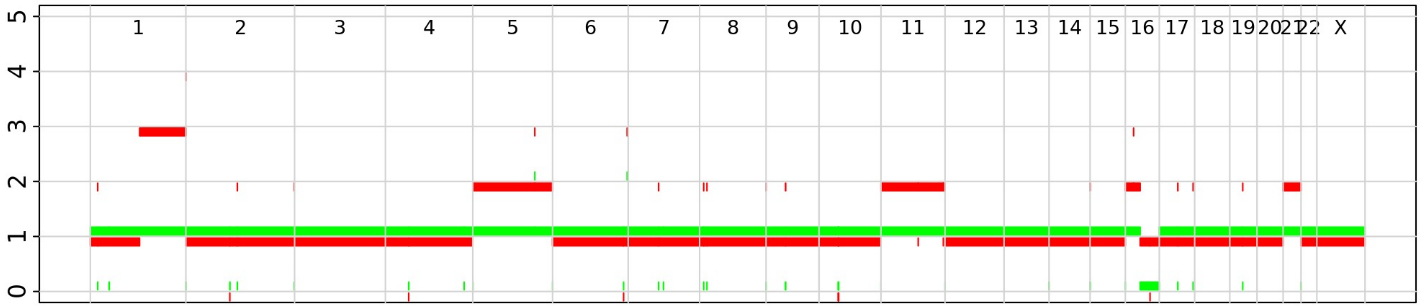

ML8 Abr

Ploidy: 3.19, aberrant cell fraction: 27%, goodness of fit: 91.4%

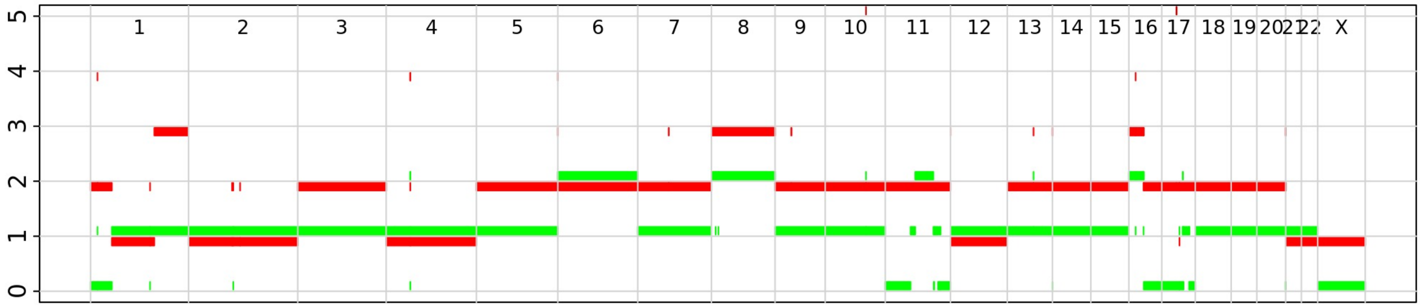

ML8 Neg

Ploidy: 1.98, aberrant cell fraction: 54%, goodness of fit: 98.0%

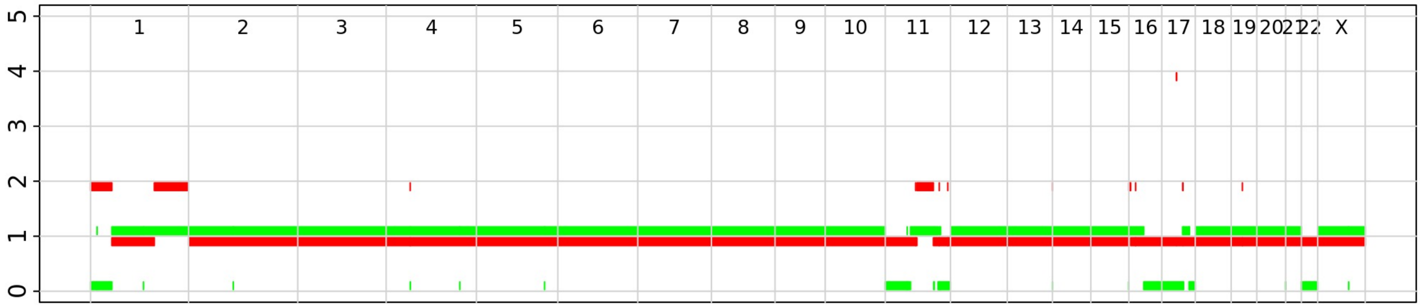

ML10 Abr

Ploidy: 4.32, aberrant cell fraction: 24%, goodness of fit: 98.5%

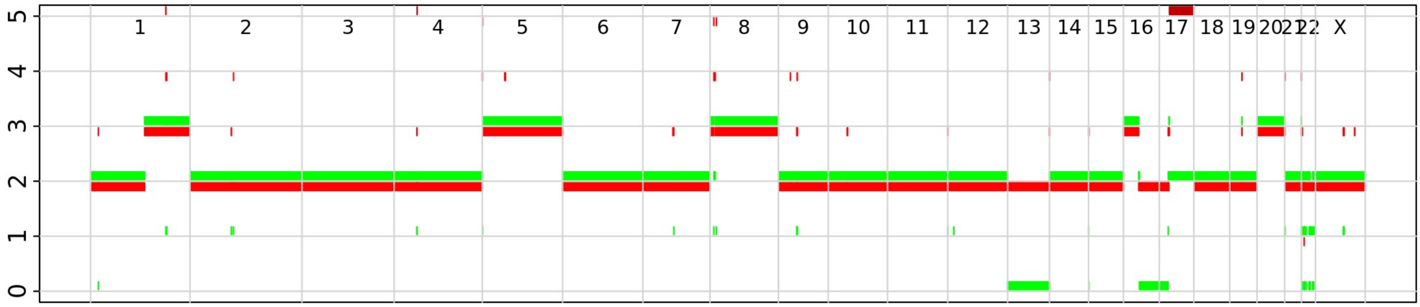

ML10 Neg

Ploidy: 2.01, aberrant cell fraction: 22%, goodness of fit: 95.7%, non-aberrant

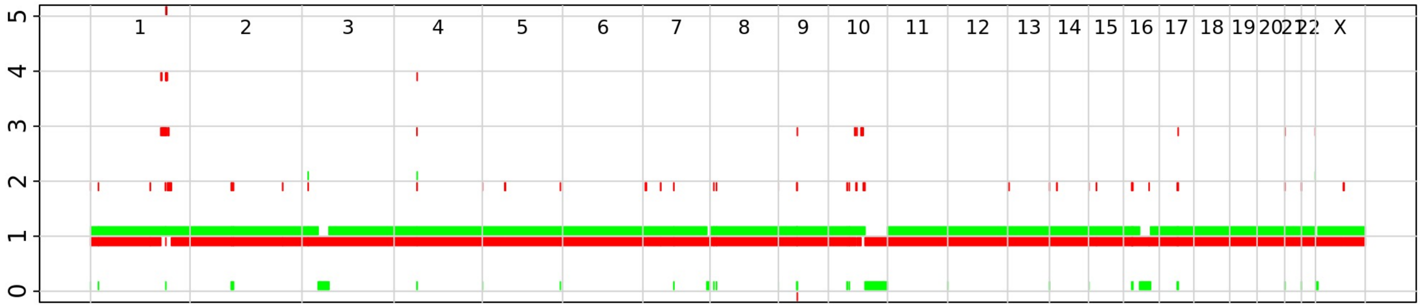

ML12 Abr

Ploidy: 2.01, aberrant cell fraction: 28%, goodness of fit: 96.1%, non-aberrant

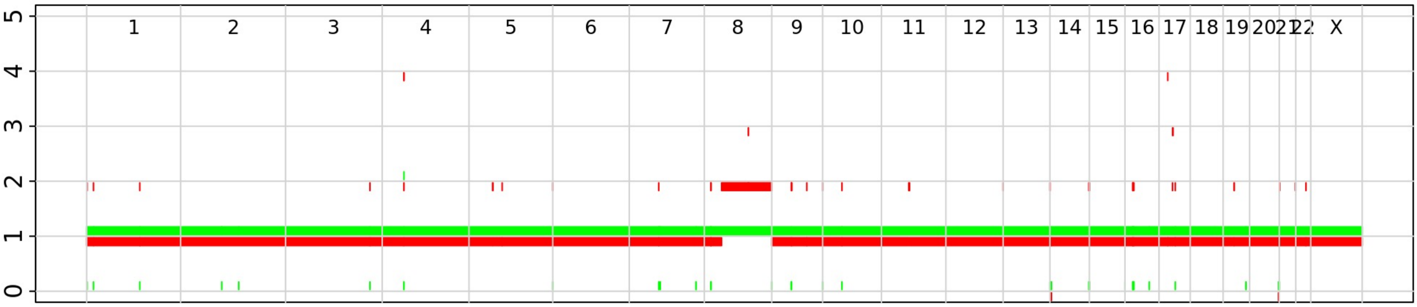

ML12 Neg

Ploidy: 2.03, aberrant cell fraction: 17%, goodness of fit: 88.8%, non-aberrant

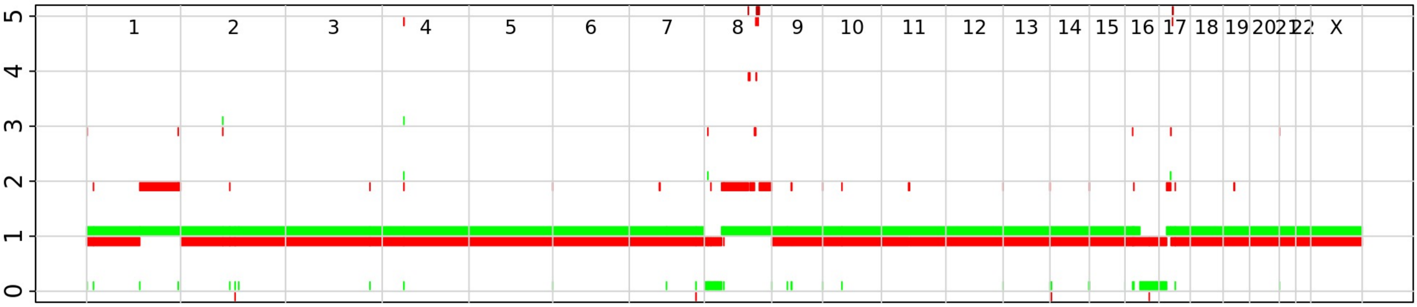

ML13 Abr

Ploidy: 1.96, aberrant cell fraction: 28%, goodness of fit: 94.5%

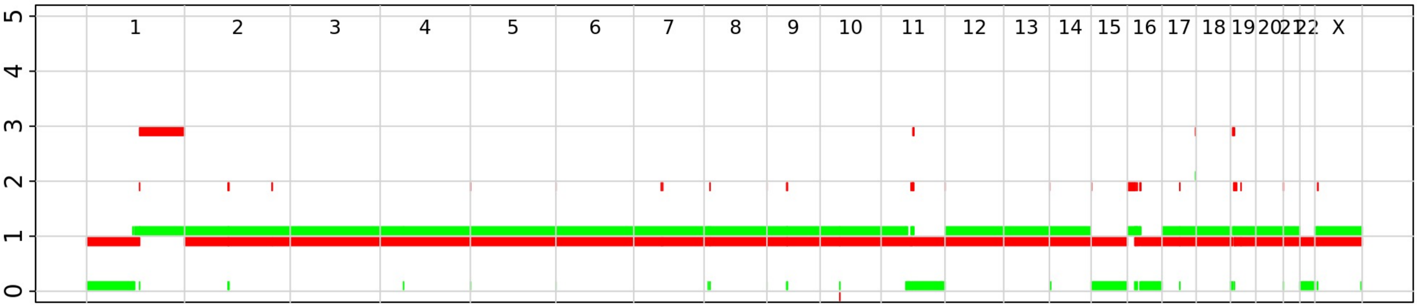

ML13 Neg

Ploidy: 1.84, aberrant cell fraction: 37%, goodness of fit: 91.5%

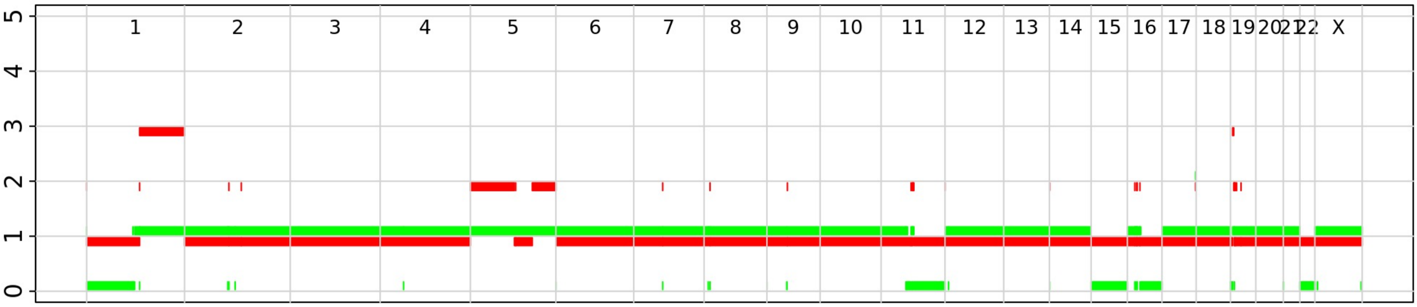

ML14 Abr

Ploidy: 2.03, aberrant cell fraction: 40%, goodness of fit: 96.2%

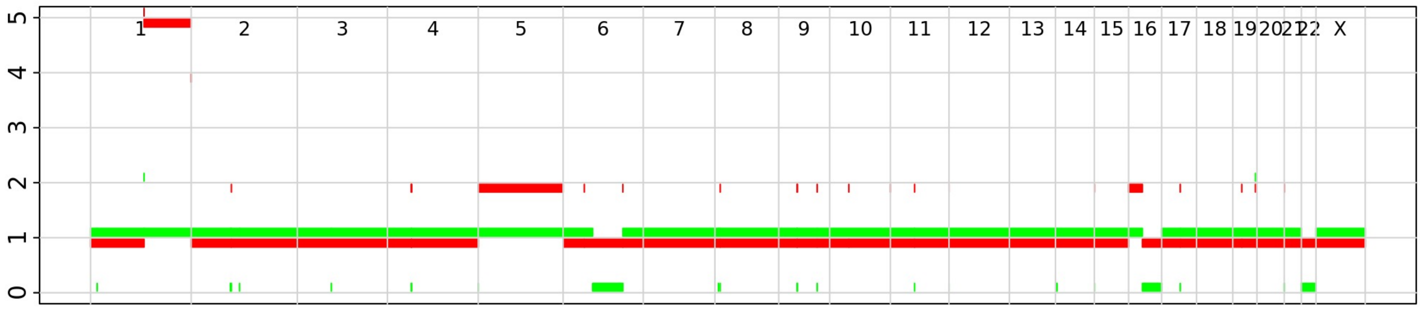

ML14 Neg

Ploidy: 2.09, aberrant cell fraction: 38%, goodness of fit: 95.2%

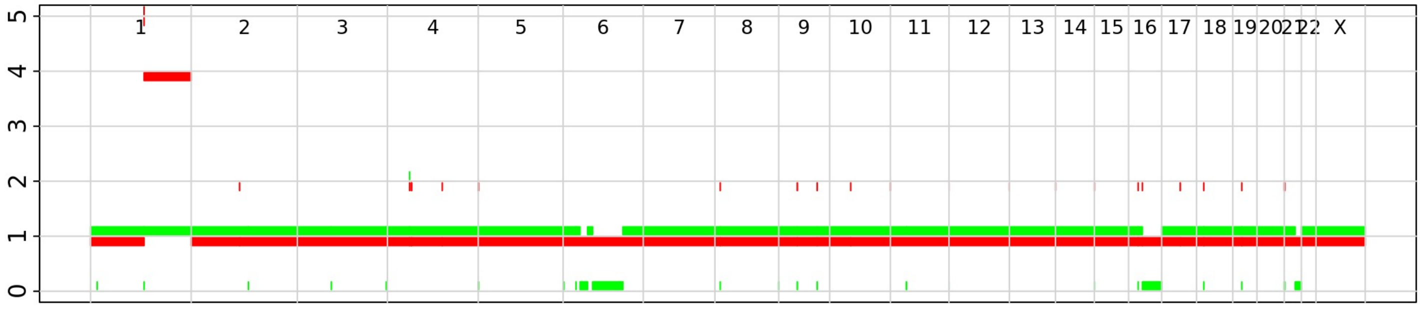

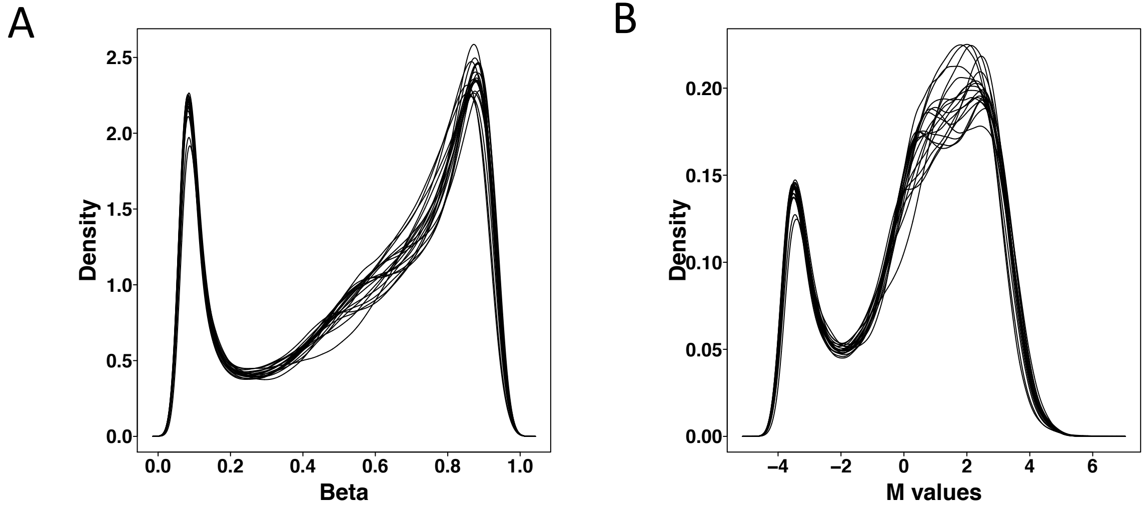

Supplementary Figure 3

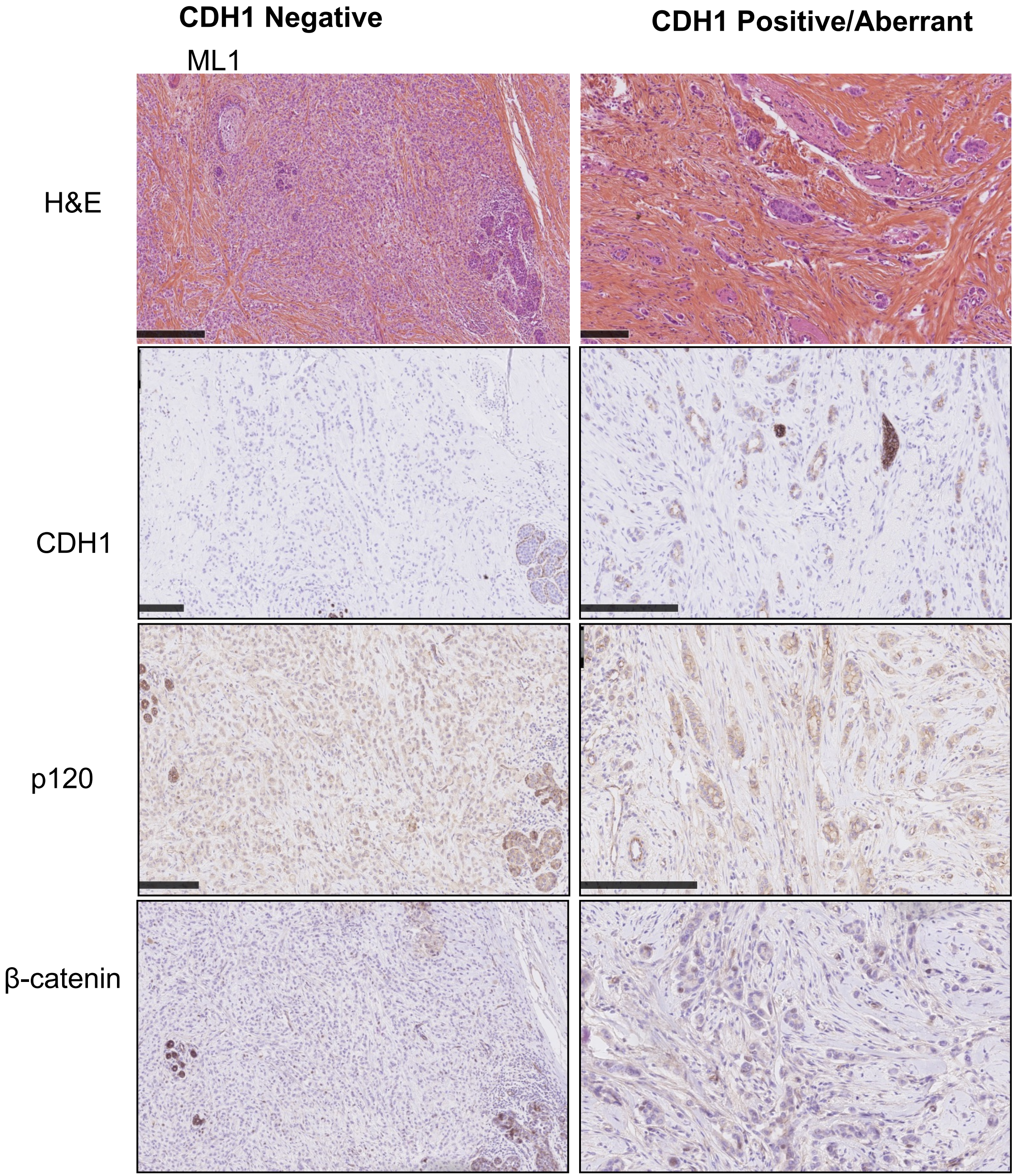

Supplementary Figure 4

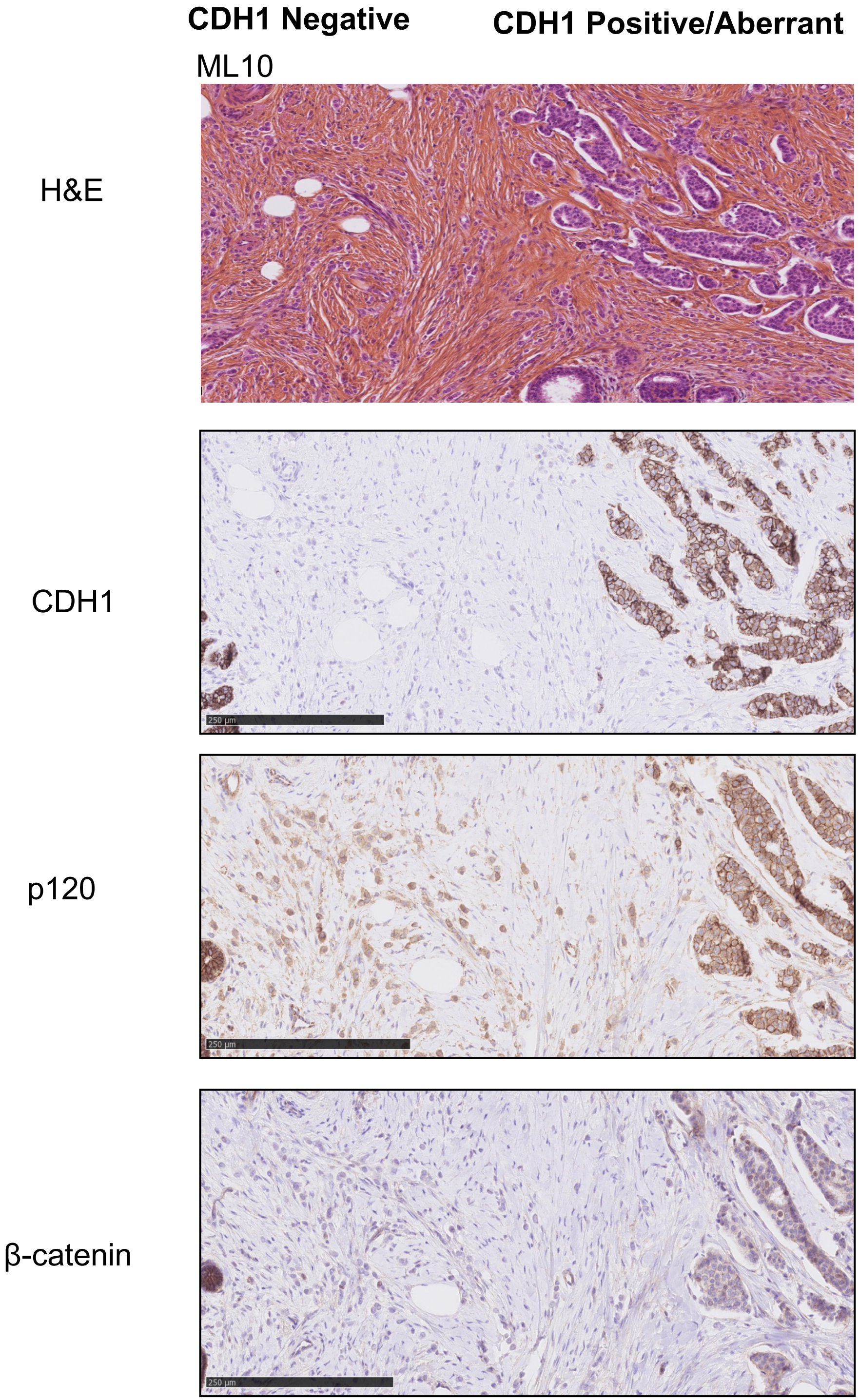

Supplementary Figure 4

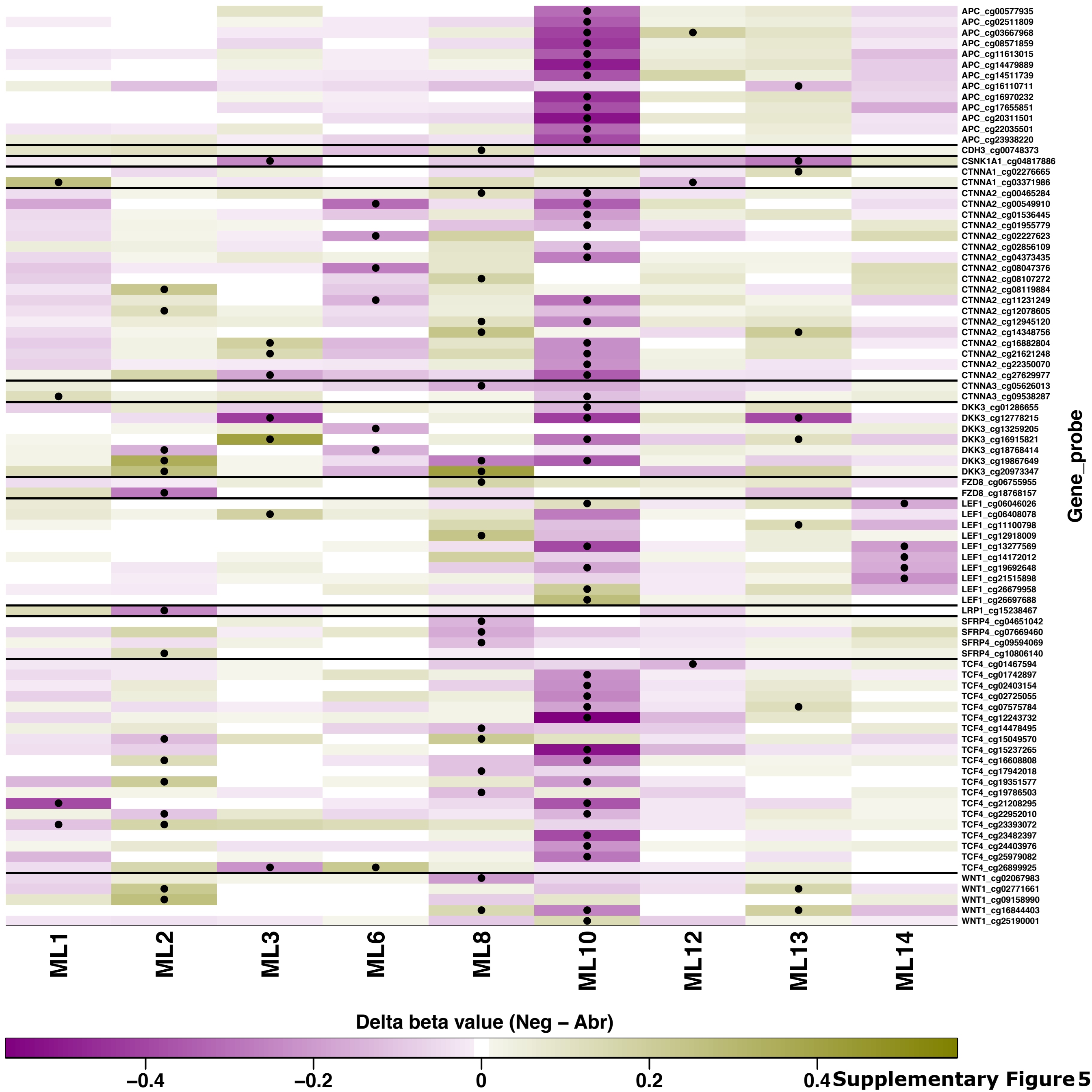

Supplementary Figure 5

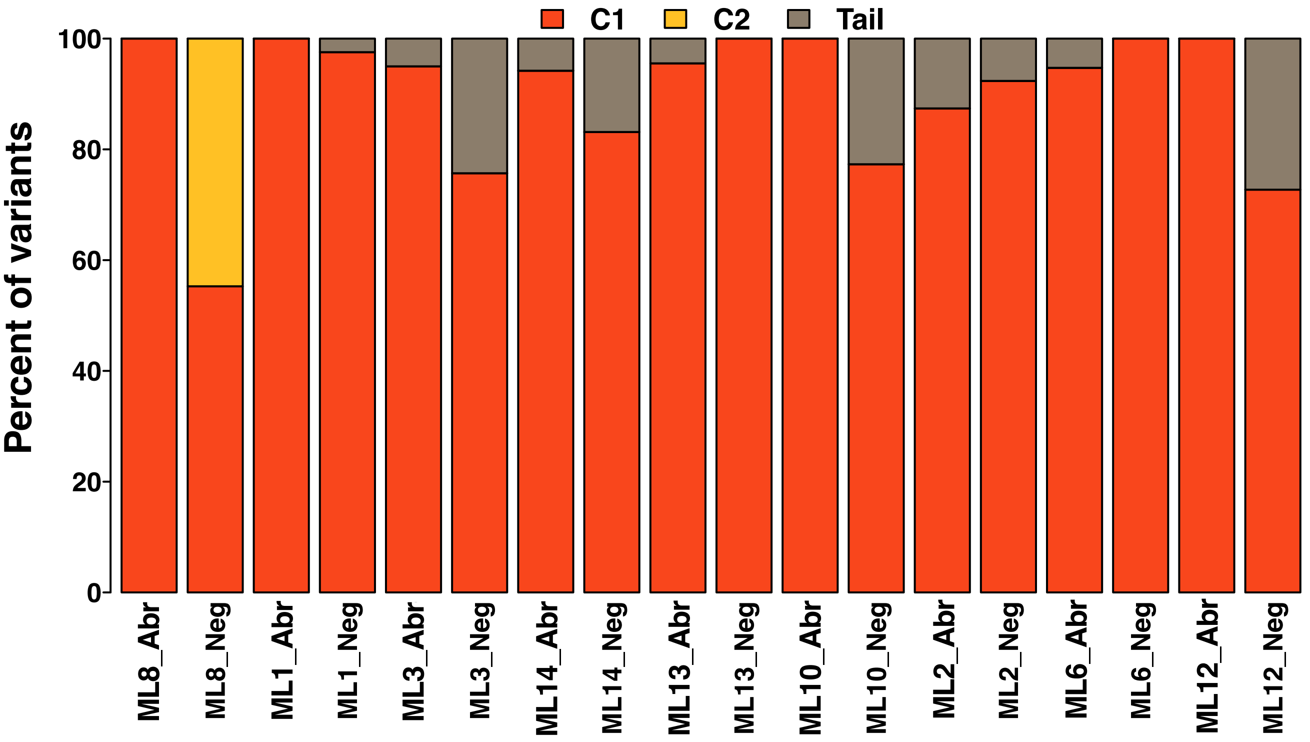

Supplementary Figure 6
